# Collagen 1 Fiber Volume Predicts for Recurrence of Stage 1 Non-Small Cell Lung Cancer

**DOI:** 10.3390/tomography10070083

**Published:** 2024-07-13

**Authors:** Samata Kakkad, Balaji Krishnamachary, Nadege Fackche, Matthew Garner, Malcom Brock, Peng Huang, Zaver M. Bhujwalla

**Affiliations:** 1 Division of Cancer Imaging Research, The Russell H. Morgan Department of Radiology and Radiological Science, The Johns Hopkins University School of Medicine, Baltimore, MD 21205, USA; samatam7@gmail.com (S.K.); bkrishn1@jhu.edu (B.K.); 2 Department of Surgery, The Johns Hopkins University School of Medicine, Baltimore, MD 21205, USA; nadege.fackche@moffitt.org (N.F.); garnerma@upstate.edu (M.G.); mabrock@jhmi.edu (M.B.); 3 Sidney Kimmel Comprehensive Cancer Center, The Johns Hopkins University School of Medicine, Baltimore, MD 21205, USA; phuang12@jhmi.edu; 4 Department of Radiation Oncology and Molecular Radiation Sciences, The Johns Hopkins University School of Medicine, Baltimore, MD 21205, USA

**Keywords:** NSCLC recurrence, SHG microscopy, collagen, extracellular matrix, tumor microenvironment

## Abstract

**Background**: The standard of care for stage 1 NSCLC is upfront surgery followed by surveillance. However, 20–30% of stage 1 NSCLC recur. There is an unmet need to identify individuals likely to recur who would benefit from frequent monitoring and aggressive cancer treatments. Collagen 1 (Col1) fibers detected by second harmonic generation (SHG) microscopy are a major structural component of the extracellular matrix (ECM) of tumors that play a role in cancer progression. **Method**: We characterized Col1 fibers with SHG microscopy imaging of surgically resected stage 1 NSCLC. Gene expression from RNA sequencing data was used to validate the SHG microscopy findings. **Results**: We identified a significant (*p* ≤ 0.05) increase in the Col1 fiber volume in stage 1 NSCLC that recurred. The increase in Col1 fiber volume was supported by significant increases in the gene expression of Col1 in invasive, compared to noninvasive, lung adenocarcinoma. Significant differences were identified in the gene expression of other ECM proteins, as well as CAFs, immune checkpoint markers, immune cytokines, and T-cell markers. **Conclusion:** Col1 fiber analysis can provide a companion diagnostic test to evaluate the likelihood of tumor recurrence following stage 1 NSCLC. The studies expand our understanding of the role of the ECM in NSCLC recurrence.

## 1. Introduction

Lung cancer is a leading cause of cancer-related deaths [1]. In the US, non-small cell lung cancer (NSCLC) accounts for approximately 85% of all lung cancers. With enhanced lung cancer screening techniques, the number of newly diagnosed stage 1 NSCLC cases is increasing [2,3,4]. Upfront surgical resection remains the gold standard for treatment of stage 1 NSCLC, with five-year overall survival rates nearing 70%. However, up to 20–30% of patients with completely resected stage 1 NSCLC are still at risk for further lung cancer development, either as a recurrence or as a new metachronous primary. In fact, recurrence is the main cause of treatment failure in these patients and the most common obstacle for long-term survival. Unfortunately, 70–90% of those who progress die from their lung cancers [5,6,7,8,9]. Neoadjuvant or adjuvant systemic therapies using targeted therapy or immunotherapy, used alone or in combination with chemotherapy, are promising approaches to improve the cure rate for patients with resectable early-stage lung cancer [10]. Early prediction of progression would allow oncologists to identify patients who may benefit from adjuvant or even immunological treatment to offset the full development of metastatic disease. Identifying patients with early-stage NSCLC who may benefit from these novel therapies is an important unmet need.

As a major component of the tumor extracellular matrix (ECM) [11], collagen 1 (Col1) fibers have been found to play an active role in cancer progression [12,13]. While significant advances have been made in understanding the role of collagen in the progression of breast [14], prostate [15], and colorectal cancer [16,17], its role in NSCLC is largely unexplored, especially in terms of differences between those early stage cancers that recur and those which do not recur. Increased Col1 fiber volumes are associated with increased likelihood of metastasis in breast cancer [12,13,18], and although the mechanism is not completely understood, studies have demonstrated a functional role for Col1 fibers in mediating of transport molecules in breast cancer xenografts [19,20]. In prostate cancer, Col1 fibers in primary cancers that metastasized were found to have patterns different from cancers that did not metastasize [15]. Similarly, in colorectal cancer, increased Col1 deposition has been positively correlated with metastasis [17]. 

Col1 fiber topography including fiber diameter [21], directionality [22], and alignment [23] plays an important role in cancer motility and invasiveness. Cancer cells can travel along aligned Col1 fibers [12,24]. Tumor-associated Col1 fiber alignment is a potential prognostic signature for survival in breast cancer patients [14]. Col1 fibers can be detected with second harmonic generation (SHG) microscopy [11] which detects an intrinsic signal derived from the non-centrosymmetric molecular structure of Col1 fibers [11,25]. In a recent study, a significant increase in Col1 fiber density as detected by SHG microscopy was observed in early-stage lung adenocarcinoma compared to normal tissue [26]. 

Our purpose here was to determine differences in Col1 fiber content in stage 1 NSCLC that subsequently recurred. We used SHG microscopy to detect and quantify Col1 fibers [18,19,20,27,28,29]. The intrinsic contrast generated by Col1 fibers with SHG microscopy allowed us to use hematoxylin and eosin (H&E)-stained sections from the National Lung Screening Trial (NLST) project of the National Institutes of Health (NIH). We have for the past decade developed analytical algorithms to characterize Col1 fibers [29]. Here we applied these algorithms to quantify differences in fiber volume in tissue sections.

To understand the causes and consequences of the increase in Col1 fiber volume observed in recurrent stage 1 NSCLC, including the immune modulatory role of collagen [30,31], we analyzed genes associated with ECM proteins including collagen, cancer associated fibroblasts (CAFs), immune checkpoints, and T-cells by mining a publicly available dataset (GSE166720) [32] from NCBI’s GEO database that contained gene expression data from noninvasive and invasive stage 1 lung adenocarcinoma. We focused on CAFs because they play an important role in the synthesis and remodeling of multiple ECM proteins including collagen [33,34,35]. CAF subtypes have been associated with establishing an immunosuppressive tumor microenvironment [36,37,38], and promoting tumor invasion and metastasis [34,35,39,40]. We analyzed immune checkpoints because of the association between CAFs and an immune suppressive tumor microenvironment in lung [41] and other cancers [33], as well as the increasing evidence of the immunomodulatory role of collagen [30,31]. Because collagen fibers have been identified as facilitating the movement of cancer cells [12,24] as well as macromolecules [20] and water movement [19], we characterized the gene expression of T-cell markers to determine if the T-cell numbers increased in invasive lung adenocarcinoma group compared to the noninvasive group. Our study identified the importance of increased collagen in lung cancer recurrence in stage 1 NSCLC that, to the best of our knowledge, has not been previously reported. Gene expression patterns identified potential causes as well as consequences of these changes in collagen that may contribute to recurrence.

## 2. Methods

### 2.1. Samples

H&E stained sections were obtained through a Material Transfer Agreement between the National Cancer Institute and Johns Hopkins University. Detailed descriptions of the NLST specimen collection and processing have been previously described [42]. We analyzed 5 µm-thick H&E stained sections from surgically resected tissue obtained from twelve patients with non-recurrent stage 1 NSCLC and fourteen patients with recurrent stage 1 NSCLC. Since the tissue sections were H&E sections from the NIH NLST database obtained during surgery, the sections were formalin fixed and mounted on glass slides and therefore did not require any special storage conditions. The ability to use H&E sections provides the advantage of including SHG microscopy analysis as a companion diagnostic. Patient demographics are presented in Table 1. All patients received informed consent for the use of their surgically resected tissues for future studies [42], and an IRB-approved waiver was obtained from the Johns Hopkins University School of Medicine.

### 2.2. SHG Imaging and Analysis

Col1 fibers were detected with SHG microscopy from the intrinsic signal derived from the non-centrosymmetric molecular structure of Col1 fibers. SHG microscopy was performed on H&E-stained tissue sections. The person performing SHG microscopy was blind to the identity of the stained tissue and the outcome of the patient. An Olympus Laser Scanning FV1000 MPE multiphoton microscope (Olympus Corp., Center Valley, PA, USA) was used to acquire the SHG signals with an excitation wavelength of 860 nm, and a detection wavelength of 430 nm, using a 25× lens, and a voxel resolution of 0.497 µm. A review of SHG microscopy and a schematic of the microscope can be found in [43]. Randomly selected fields of view (FOVs) ranging from 6–18 FOVs from each tissue block, one block per patient, were analyzed. The mean fiber volume for each patient in the non-recurrent and recurrent group, obtained from 6–18 randomly selected FOVs, was used for statistical analysis. We also analyzed individual FOVs using a random effects model. 

We performed tiled scan SHG microscopy on six sections (three non-recurrent and three recurrent patients) to acquire the Col1 fiber distribution over the entire tissue sample. Tiled scan SHG microscopy of the entire tissue sample was performed using a 25× lens, at a voxel resolution of 0.53 μm × 0.53 μm, and at z-intervals of 3 μm. Tiled scan acquisitions of the entire biopsied section were divided into smaller sections. The biopsy tissue sections ranged from 1.5 cm × 1.5 cm to 1.5 cm × 2.2 cm that were beyond the data holding capacity of the microscope software optical signal limits of 0.7 cm × 0.7 cm at the set resolution of 0.53 μm × 0.53 μm and z-intervals of 3 μm. We therefore divided each tissue section into 0.7 cm × 0.7 cm square quadrants. SHG signal was acquired from each of these quadrants with ~300 μm of overlapping regions with the neighboring quadrants. Once the SHG data from each of the quadrants were acquired for a tumor section, an in-built MATLAB R2017b code (MathWorks Inc., Natick, MA, USA) was used to stitch the quadrants together to overlay the SHG information on the corresponding H&E section. 

Col1 fibers in sections were quantified to calculate the percent fiber volume using an in-built MATLAB R2017b code (MathWorks Inc., Natick, MA, USA). The quantification analysis was done as previously described [18,19,20,27]. Briefly, Col1 fibers were extracted using fuzzy c-mean clustering segmentation and applying length and width criteria to filter out stray signals, to quantify percent fiber volume as previously described [18,19,20,27]. Our software quantified the total Col1 fiber volume by first preprocessing the raw image to exclude noise and nonfibrillar shapes by using a shape filter as previous described [27]. The Col1 fiber structures extracted from the raw images were analyzed as the percent Col1 fiber volume per field of view.

### 2.3. Molecular Analysis of Noninvasive and Invasive Lung Adenocarcinoma

We analyzed genes associated with ECM proteins, CAFs, immune checkpoints, and T-cell markers to further understand the potential causes and impact of the Col1 changes observed with SHG microscopy in our study, using a publicly available dataset that contained gene expression data from 32 noninvasive and 21 invasive stage IA lung adenocarcinoma, classified based on gene expression signatures [32]. We mined this publicly available dataset (GSE166720) [32], which was retrieved from the GEO database and analyzed using in-built software GEO2R (https://www.ncbi.nlm.nih.gov/geo/geo2r/, accessed on 25 August 2023) [44]. The interactive web tool GEO2R is based on the R programing language with an in-built statistical program and graphic tools that allow identification of differentially expressed genes. We analyzed differences in genes associated with ECM proteins, CAFs, immune checkpoints, and T-cell markers in the noninvasive and invasive samples to further understand the potential causes and consequences of the Col1 changes observed with SHG microscopy in our study. Genes expressed with at least >0.5 or <0.5 log2 fold change (~1.4-fold change) and a *p*-adjusted value (padj) of ≤0.05 were considered significantly altered.

### 2.4. Statistical Analysis

The data were expressed as mean ± SE. We used a one-tailed unpaired Student t-test as we hypothesized the recurrent tumors would have a denser collagen 1 fiber distribution compared to the non-recurrent tumor patient groups based on previously published data with several other cancers, including a recently published study with lung cancer that showed ncreased Col1 fibers in early-stage lung cancer compared to normal tissue [26]. We used a *t*-test since the data did not seriously violate the normality distribution assumption. We also performed an analysis of the percent fiber volume using individual values from the randomly selected fields of view (FOVs) ranging from 6–18 FOVs from each tissue block, with one block per patient analyzed. To compare the percent fiber volume between patients with and without recurrence, we employed a random effects model. In this model, the tissue block served as the random effect (random intercept), while the cancer recurrence status was the fixed effect. Although the mean percent fiber volume from all FOVs from the same tissue block is approximately normally distributed, the individual percent fiber volumes across all FOVs are skewed. We thus log-transformed the individual FOV percent fiber volume to reduce skewness before fitting the random effects model. Values of *p* <  0.05 were considered significant, unless otherwise stated. 

## 3. Results

**Figure 1:**
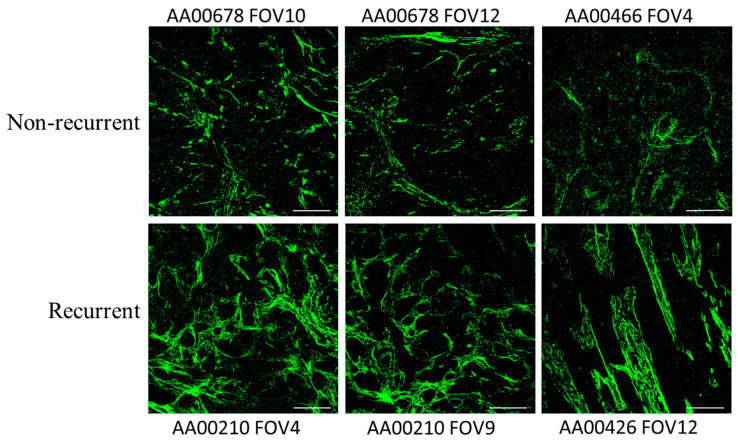
Representative SHG microscopy images showing Col1 fibers from three different non-recurrent and recurrent NSCLC tumors. SHG images were acquired with a FOV = 423.5 μm × 423.5 μm, pixel resolution in XY plane = 0.414 μm. Scale bar = 100 μm. The corresponding file names of the de-identified data are provided together with the images. The images represent Col1 fibers from two different patients with non-recurrent tumors and two different patients with recurrent tumors.

**Figure 2:**
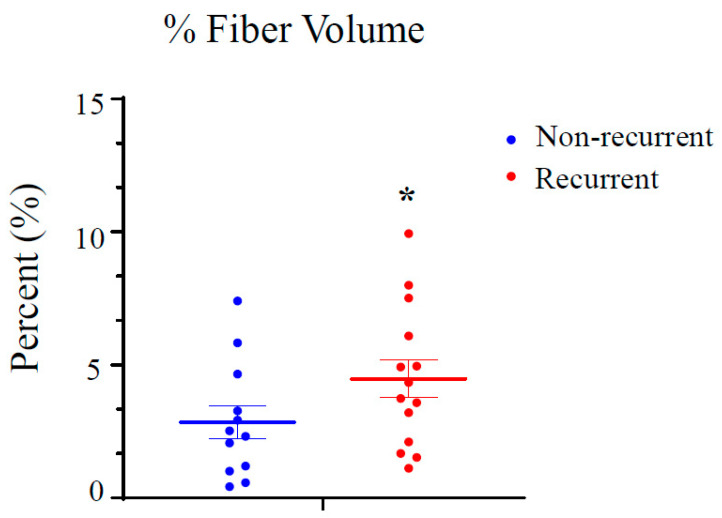
Significantly higher percent Col1 fibers were observed in the recurrent NSCLC (N = 14) compared to the non-recurrent NSCLC (N = 12). Values represent mean ± S.E.; * *p*-value ≤ 0.05. Each point represents the mean fiber volume for each patient in the non-recurrent and recurrent group obtained from 6–18 randomly selected FOVs for each patient.

Representative images of Col1 fibers acquired using SHG microscopy from three patients in the recurrent and non-recurrent groups of the NIH NLST dataset are presented in Figure 1. Increased Col1 fibers are evident in the recurrent group compared to the non-recurrent group. The quantification of the Col1 fibers identified a significant increase (*p*-value < 0.05) in Col1 fiber volume in recurrent NSCLC (N = 14) compared to the non-recurrent NSCLC (N = 12), as shown in Figure 2. The mean fiber volume for each patient was obtained from 6–18 randomly selected FOVs per patient. Each point in Figure 2 represents the mean fiber volume for each patient in the non-recurrent and recurrent group. As evident in this figure, there was some overlap between the mean fiber volume detected in the recurrent and non-recurrent group. Individual FOVs displayed for the two groups, together with the corresponding statistical analysis, are presented in Supplementary Figure S1. The model output indicated that recurrent patients exhibited a higher percent fiber volume than non-recurrent patients (two-sided test *p*-value = 0.078, one-sided test *p*-value = 0.039).

**Figure 3:**
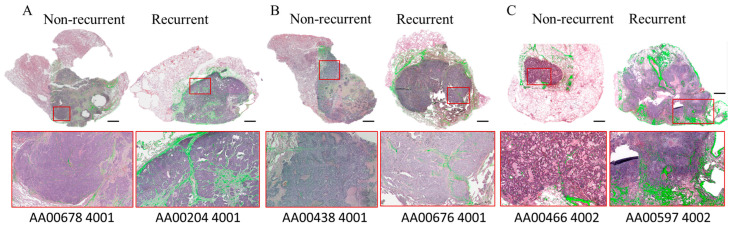
(**A**–**C**): Representative H&E stained sections and tile scanned Col1 fiber SHG microscopy images (shown in green) from three pairs of different non-recurrent and recurrent NSCLC patients, with the corresponding de-identified file names. Expanded red boxed regions identify the long thicker patterns of Col1 fiber in the H&E section of the recurrent tumors compared to the short thin Col1 fibers in the non-recurrent tumors. Pixel resolution in XY plane = 0.53 μm. Scale bar = 2000 μm.

We also performed tiled scan SHG microscopy on six of these surgical biopsy samples (three non-recurrent and three recurrent samples) to detect the Col1 fiber distribution within the entire section. This tiled scans dataset confirmed the increased Col1 fiber volume identified when analyzing FOVs. Tiled scanning also revealed thick long fiber tracks throughout the tumor regions in the recurrent NSCLC compared to a short and less dense fiber distribution in the tumor regions of the non-recurrent NSCLC as shown in Figure 3.

**Figure 4:**
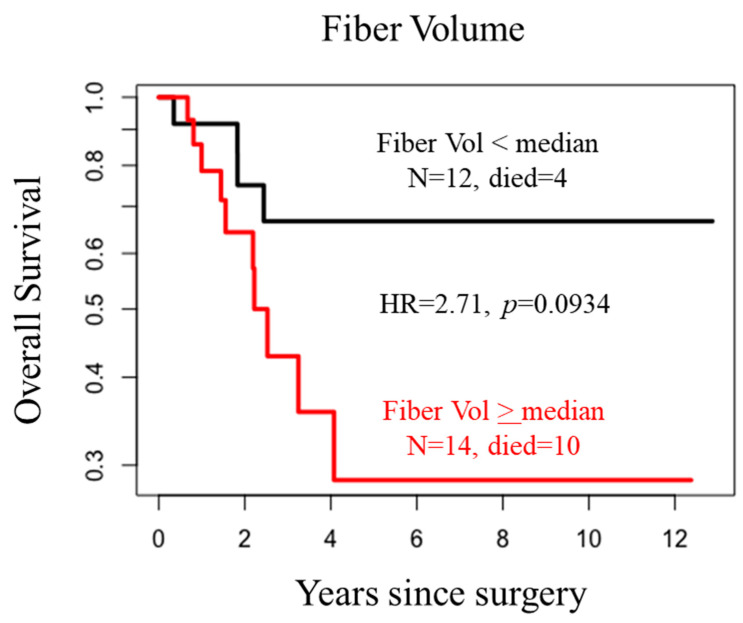
Survival graphs showing differences in the overall survival of patients from the date of surgery differentiated based on Col1 fiber volume. Patients were classified into two subgroups based on whether the Col1 fiber volume values were greater or smaller than the median value. A hazard ratio (HR) of 2.71 was observed between the two groups.

We next performed a ranked-test to predict survival in patients relative to the Col1 fiber volume. Patients were classified into two subgroups based on whether the Col1 fiber volume values were greater or smaller than the median. As shown in Figure 4, a separation was observed (*p*-value = 0.093, HR = 2.71), in terms of overall survival from time of surgery, based on the Col1 fiber volume. 

An analysis of the gene expression data from the GSE166720 dataset from the GEO database are presented in Table 2 and Table 3. The gene expression of ECM-related proteins that were significantly altered between noninvasive and invasive lung adenocarcinoma are presented in Table 2. The most prominent increase in gene expression in the invasive group was observed for Col1, followed by fibronectin. Increases in laminin, nidogen-2, and aggrecan gene expression were also observed. A significant decrease was observed for hyaluronan binding protein-2, in the invasive group compared to the noninvasive group. We also analyzed gene expression patterns of CAF markers. As shown in Table 2, a significant increase in the gene expression of multiple CAF markers associated with different subsets of CAFs, including fibroblast activation protein-alpha (FAP-α) expressing CAFs, was observed in invasive lung adenocarcinoma compared to noninvasive lung adenocarcinoma.

To identify potential alterations in immune checkpoints and T-cell markers associated with a high Col1 phenotype, we characterized gene expression patterns of immune checkpoints and T-cell markers as shown in Table 3. Several immune checkpoints including PD-1 and PD-L1 were significantly higher in the invasive lung adenocarcinoma group compared to the noninvasive group as shown in Table 3. Gene expression of cytokines that induce an immune response, as well as multiple T-cell markers, significantly increased in invasive lung adenocarcinoma. Interferon-gamma gene expression was three-fold higher in the invasive lung adenocarcinoma group. T-cell markers included those associated with cytotoxic T cells, helper T cells, memory T cells, tissue resident memory T cells, recent thymic emigrants, tissue effector memory cells, and T effector cells.

## 4. Discussion

We identified significant differences in the Col1 fiber volume between recurrent and non-recurrent stage 1 NSCLC in this exploratory study. These data highlight the potential role of Col1 fibers in the recurrence of stage 1 NSCLC and support further investigation of their use as a companion diagnostic marker to identify patients with early-stage NSCLC who may benefit from treatment. Consistent with the increase of Col1 fibers in recurrent early-stage NSCLC, Col1 fiber volume was a determinant in overall survival in these patients.

Mining the gene expression patterns of a publicly available dataset for ECM proteins, CAF markers, immune checkpoints, and T-cells provided insight into potential mechanisms underlying why Col1 fibers should have such a strong correlation with recurrence and survival. Although the gene expression data was a comparison between stage I noninvasive and locally invasive lung adenocarcinoma rather than recurrent NSCLC, it provided insight into the role of the ECM, CAFs, immune checkpoints, and immune cells in lung adenocarcinoma progression. The results from this publicly available dataset identified a significant increase in genes associated with the ECM proteins, with the largest fold increase observed for Col1 in the invasive group. Increases in the gene expression of fibronectin, laminin, nidogen-2, and aggrecan were also observed, together with a significant decrease of hyaluronan binding protein in the invasive group. Taken together, these data indicate that invasive NSCLC can significantly reprogram the ECM. Increased fibronectin [48], laminin [49,50], nidogen [51], and aggrecan [52] have been previously associated with an aggressive phenotype and progression in lung cancer. Collagens type I, II, and III form a major component of the tumor ECM, with collagen type IV lining the basement membrane and forming a network to act as a barrier to cancer cell invasion [53]. Fibronectin is frequently upregulated in many invasive tumors, including NSCLC, and undergoes post-translational modification resulting in binding to various ECM proteins. Fibronectin decorates linearized Col1, playing a role in the directional migration of tumor cells towards the vasculature for intravasation [54]. Laminins contribute to the structure of the basement membrane by binding to type IV collagen and strengthening the basement lamina [55]. In early stages of NSCLC, nidogen-2 increase is predicted to be a marker of a poor prognosis [56]. The interaction between collagen type-IV, laminin, and nidogen-2 is critical for cell adhesion, migration, and proliferation. Aggrecans are proteoglycans that play a role in cancer tissue mechanics [48]. 

Surprisingly, a five-fold reduction was observed in the gene expression of hyaluronan binding protein 2 (HABP2). HABP2 is a serine protease that binds to hyaluronic acid and modulates the ECM by accelerating the matrix degradation that eventually affects migration, extravasation, tumor growth, and metastasis [57]. HAPB2 has been previously reported to be an important regulator of lung cancer progression and its expression has been observed in NSCLC [57]. These differences suggest that the increase in HAPB2 proteins observed in NSCLC may be post-translational.

We were able to corroborate, independently, the increase of Col1 fibers identified using SHG microscopy from the H&E-stained slides from the NLST dataset with the six-fold increase of Col1 gene expression in the GEO database. We were, however, unable to map the remaining changes in the gene expression of ECM proteins, CAFs, immune checkpoints, immune cytokines, and T-cells to protein expression or immunohistochemistry due to the unavailability of tissues, which was a limitation of this study. 

We used known molecular markers associated with CAFs to screen for differences between the invasive and noninvasive groups. Changes in gene expression of CAF markers were associated with several CAF subsets [45]. The molecular markers that showed a significant difference between the two groups were associated with antigen-presenting CAFs (apCAFs), inflammatory CAFs (iCAFs), myofibroblast CAFs (myCAFs), and FAP-α CAFs. Notably, in lung cancer [41], as well as in multiple other cancers [33], FAP-α expressing CAFs have been associated with fibrogenesis and with immune suppression. An increase of FAP-α CAFs may explain the increase of Col1 fibers in recurrent NSCLC.

A significant increase in the gene expression of several immune checkpoints, including PD-L1 and PD-1, was observed in the invasive lung adenocarcinoma group, suggestive of an immune suppressive microenvironment [58,59]. The gene expression of immune cytokines such as interferon gamma, as well as different T-cell markers, also significantly increased, consistent with previously published reports [60,61].

The SHG microscopy data provided clear evidence that Col1 fibers significantly increased in recurrent stage 1 NSCLC in this exploratory study. While a previous study has reported a significant increase in Col1 fiber density, as detected by SHG microscopy in early-stage lung adenocarcinoma compared to normal tissue [26], here we found that Col1 fibers increased significantly in recurrent compared to non-recurrent stage 1 NSCLC. Expanded future studies with a large data base would be required to identify a Col1 fiber threshold value for risk of recurrence of stage 1 NSCLC. Future expanded studies should also investigate a more in-depth geometric and textural analysis, and evaluate polarization-resolved measurements in normal tissue, as well as stage 1 NSCLC. Col1 fibers mediate the movement of molecules and cells through the ECM [12,15,19,20]. Lung cancer recurrences from patients in this study were both distant and local, supporting the possibility that the fibers may have mediated the migration of cancer cells to distant or local sites. From our collective data, we hypothesize that collagen may also mediate an increase of T-cell movement into the tumor by providing transport pathways. This may be counter balanced by immunomodulation of T-cells by collagen [30,31] and CAFs resulting in T-cell exhaustion as evident from the increase of immune checkpoint expression.

## 5. Conclusions

Our study highlights the importance of the tumor ECM and microenvironment in NSCLC recurrence. Our observations linking Col1 fiber volume to cancer progression in post-operative stage 1 NSCLCs identify Col1 fibers as potential biomarkers with clinical significance. Col1 fiber analysis may provide a companion diagnostic test that can be performed rapidly on H&E tumor sections, using nondestructive SHG microscopy, to evaluate the likelihood of tumor recurrence from stage I NSCLC. The studies here also expand our understanding of the role of the ECM in NSCLC recurrence that may lead to new targets for future therapeutic strategies. Future studies should characterize the association between Col1 fibers and the migration of cancer cells. The role of Col1 and ECM proteins in the modulation of immune checkpoints in NSCLC should also be investigated.

## Figures and Tables

**Table 1:** Demographics of the patient population studied.

		No Recurrence	* Recurrence	*p* Value
N		12	14	
Female	N (%)	4 (33%)	7 (50%)	1.0
Age at surgery	Mean ± sd	63.3 ± 4.4	66.5 ± 5.4	0.1141
Current smoker	N (%)	4 (33%)	10 (71%)	0.1131
Years smoked	Mean ± sd	42.0 ± 6.5	47.9 ± 6.6	0.0326
Pack-years	Mean ± sd	55.0 ± 17.0	83.3 ± 63.7	0.1308
Family history of lung cancer	N (%)	2 (17%)	5 (36%)	0.3913

* 8 local, 3 distant, 3 local and distant.

**Table 2:** Log2 fold changes in ECM-protein-related genes in invasive compared to noninvasive NSCLC samples in the GSE166720 data set. Log2 fold negative values represent downregulated genes.

	Description	Symbol	GeneID	log2Fold Change	*p* Value	padj
**ECM related proteins**	Collagen type I alpha 1 chain	COL1A1	1277	2.63	5.27 × 10^−19^	0.0000
	Fibronectin type III domain containing 1	FNDC1	84,624	1.97	2.44 × 10^−11^	0.0000
	Collagen type I alpha 2 chain	COL1A2	1278	1.93	3.44 × 10^−14^	0.0000
	Laminin subunit beta 3	LAMB3	3914	1.64	4.20 × 10^−10^	0.0000
	Laminin subunit gamma 2	LAMC2	3918	1.62	5.88 × 10^−07^	0.0000
	Nidogen 2	NID2	22,795	1.51	2.04 × 10^−13^	0.0000
	Aggrecan	ACAN	176	1.50	3.76 × 10^−05^	0.0003
	EGF-like, fibronectin type III and Laminin G domains	EGFLAM	133,584	1.39	3.38 × 10^−12^	0.0000
	Laminin subunit beta 4	LAMB4	22,798	1.36	6.52 × 10^−06^	0.0001
	Laminin subunit alpha 1	LAMA1	284,217	0.75	7.09 × 10^−03^	0.0228
	Laminin subunit beta 1	LAMB1	3912	0.73	4.16 × 10^−05^	0.0003
	Laminin subunit alpha 4	LAMA4	3910	0.52	2.84 × 10^−03^	0.0108
	Hyaluronan binding protein 2	HABP2	3026	−2.37	8.10 × 10^−07^	0.0000
**CAFs**	HHIP like 2 (myCAF) [45]	HHIPL2	79,802	3.27	1.44 × 10^−14^	0.0000
	Leucine-rich repeat containing 15 (myCAF)	LRRC15	131,578	2.37	6.07 × 10^−10^	0.0000
	Thy-1 cell surface antigen (myCAF) [45,46]	THY1	7070	1.44	1.02 × 10^−13^	0.0000
	Fibroblast activation protein alpha	FAP	2191	1.40	5.33 × 10^−09^	0.0000
	HHIP like 1 (myCAF) [45]	HHIPL1	84,439	0.85	2.06 × 10^−05^	0.0002
	Platelet derived growth factor receptor beta (iCAF) [46]	PDGFRB	5159	0.55	9.45 × 10^−04^	0.0044
	Integrin subunit alpha 8 (apCAF) [46]	ITGA8	8516	−0.82	3.29 × 10^−05^	0.0003

apCAF: antigen-presenting CAF; iCAF: inflammatory CAF; myCAF: myofibroblasts CAF.

**Table 3:** Log2 fold changes in immune checkpoint and T-lymphocyte related genes in invasive compared to noninvasive NSCLC samples in the GSE166720 data set. Log2 fold negative values represent downregulated genes.

	Description	Symbol	GeneID	log2Fold Change	*p* Value	padj
**Immune checkpoints**	Lymphocyte activating 3	LAG3	3902	1.62	2.81 × 10^−10^	0.0000
	Indoleamine 2,3-dioxygenase 1	IDO1	3620	1.60	2.73 × 10^−06^	0.0000
	Sialic acid binding Ig like lectin 10	SIGLEC10	89,790	1.35	4.07 × 10^−07^	0.0000
	V-set domain containing T cell activation inhibitor 1	VTCN1	79,679	1.33	1.30 × 10^−02^	0.0373
	Programmed cell death 1	PDCD1	5133	1.09	2.36 × 10^−05^	0.0002
	CD274 molecule	CD274	29,126	0.68	1.55 × 10^−02^	0.0430
**Immune cytokines/** * T-lymphocytes	Interferon gamma (Th1, Tc1)	IFNG	3458	1.80	1.32 × 10^−05^	0.0001
	Transforming growth factor beta induced (Tregs)	TGFBI	7045	1.11	1.06 × 10^−08^	0.0000
	CD8b molecule (Tc)	CD8B	926	0.85	1.69 × 10^−03^	0.0070
	Interleukin 2 receptor subunit alpha (Tregs, Tcm)	IL2RA	3559	0.84	3.54 × 10^−03^	0.0129
	Integrin subunit alpha E (Trm, RTE)	ITGAE	3682	0.82	4.69 × 10^−07^	0.0000
	CD38 molecule (activated T cells)	CD38	952	0.71	2.01 × 10^−02^	0.0531
	CD8a molecule (Tc)	CD8A	925	0.69	7.49 × 10^−03^	0.0238
	Interleukin 10 (Tregs, Trm, Th2, Tc9)	IL10	3586	0.68	6.95 × 10^−03^	0.0224
	CD3 delta subunit of T-cell receptor complex (Pan T cells)	CD3D	915	0.60	6.17 × 10^−03^	0.0204
	Interleukin 2 receptor subunit beta (Tscm, Tcm, Tem, Teff)	IL2RB	3560	0.59	1.21 × 10^−02^	0.0351
	C-C motif chemokine receptor 6 (Th17, Tc17)	CCR6	1235	−0.59	3.2 × 10^−03^	0.0119
	prostaglandin D2 receptor 2 (Th2, Tc2)	PTGDR2	11,251	−0.72	1.20 × 10^−02^	0.0348
	CD69 molecule (Trm, Tcm, Tem, Teff)	CD69	969	−0.72	2.08 × 10^−03^	0.0083

RTE: Recent thymic emigrants; Tc: Cytotoxic T cells; Tcm: Memory T cells; Teff: Effector T cells; Tem: Tissue effector memory cells; Th1: Helper T cells; Tregs: Regulatory T cells, Th2: Helper T cells; Trm: Tissue resident memory T cells; Tscm: memory stem cell; * Abbreviations from [47].

## Data Availability

Data will be shared upon reasonable request from the corresponding author.

## Supplementary Materials

The following supporting information can be downloaded at: https://www.mdpi.com/article/10.3390/tomography10070083/s1, Figure S1: Percent fiber volume from randomly selected fields of view (FOVs) ranging from 6-18 FOVs from each tissue block, one block per patient were analyzed. To compare the percent fiber volume between patients with and without recurrence, we employed a random effects model. In this model, the tissue block served as the random effect (random intercept), while the cancer recurrence status was the fixed effect. Additionally, we log-transformed the percent fiber volume to reduce skewness before fitting the model. The model output indicated that recurrent patients exhibited a higher percent fiber volume than non-recurrent patients (two-sided test *p*-value = 0.078, one-sided test *p*-value = 0.039).

## Abbreviations

CAFs	Cancer-associated fibroblasts
Col1	Collagen 1
ECM	Extracellular matrix
FOVs	Fields of view
GEO	Gene expression omnibus
H&E	Hematoxylin and eosin
IRB	Institutional Review Board
NCBI	National Center for Biotechnology Information
NIH	National Institutes of Health
NLST	National Lung Screening Trial
NSCLC	Non-small cell lung carcinoma
SHG	Second harmonic generation
