# Supplementary material for: Collagen 1 Fiber Volume Predicts for Recurrence of Stage 1 Non-Small Cell Lung Cancer
Source: Tomography. 2024 Jul 13;10(7):1099–112. doi: 10.3390/tomography10070083 (PMC11281282; doi:10.3390/tomography10070083)
Supplement: Supplementary file 1 [file tomography-10-00083-s001.zip › tomography-2992364-supplementary.pdf]

Supplementary Figure S1

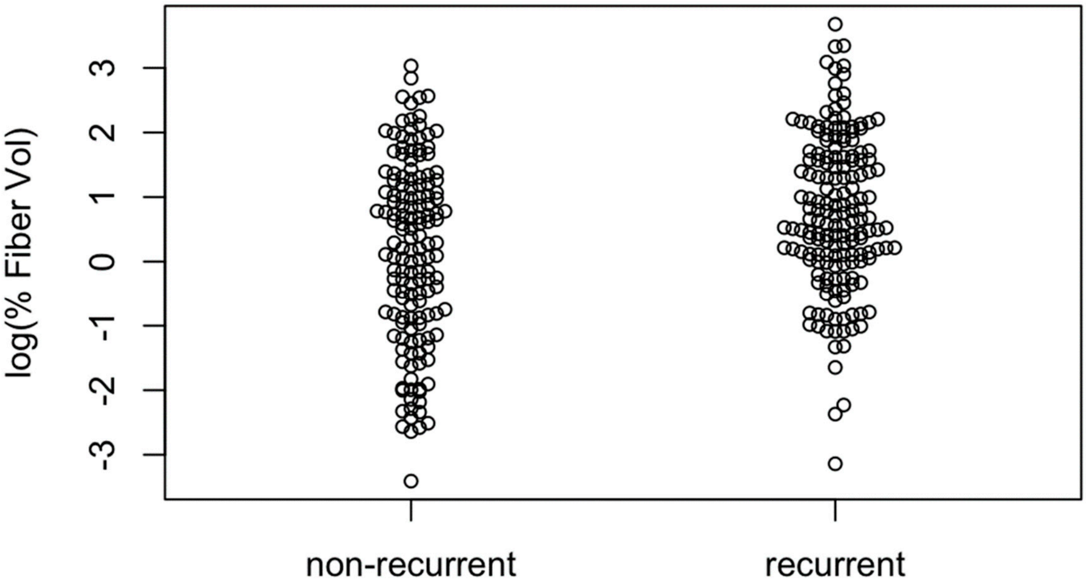

Supplementary Figure S1: Percent fiber volume from randomly selected fields of view (FOVs) ranging from 6-18 FOVs from each tissue block, one block per patient were analyzed. To compare the percent fiber volume between patients with and without recurrence, we employed a random effects model. In this model, the tissue block served as the random effect (random intercept), while the cancer recurrence status was the fixed effect. Additionally, we log-transformed the percent fiber volume to reduce skewness before fitting the model. The model output indicated that recurrent patients exhibited a higher percent fiber volume than non-recurrent patients (two-sided test p-value = 0.078, one-sided test p-value = 0.039).
